# Development and evaluation of artificial organ models for ERCP training in patients with surgically altered anatomies

**DOI:** 10.1038/s41598-023-49888-3

**Published:** 2023-12-21

**Authors:** Kai Koch, Benedikt Duckworth-Mothes, Ulrich Schweizer, Karl-Ernst Grund, Tom G. Moreels, Alfred Königsrainer, Dörte Wichmann

**Affiliations:** 1 Working Group of Experimental Endoscopy, Development, and Training, University Hospital Tübingen, Waldhörnlestrasse 22, 72072 Tübingen, Germany; 2 Department of Gastroenterology and Hepatology Klinikum Neuperlach, Oskar-Maria-Graf-Ring 51, 81737 Munich, Germany; 3 Department of General, Visceral and Transplantation Surgery, University Hospital of Tübingen, Hoppe-Seyler-Str. 3, 72076 Tübingen, Germany; 4 Department of Gastroenterology and Hepatology, Cliniques Universitaires Saint-Luc, Av. Hippocrate 10, 1200 Brussels, Belgium; 5 Central Endoscopy, University Hospital of Tübingen, Otfried-Müller-Str. 10, 72076 Tübingen, Germany

**Keywords:** Gastrointestinal diseases, Gastrointestinal models, Gastrointestinal system

## Abstract

Endoscopy training models (ETM) using artificial organs are practical, hygienic and comfortable for trainees. However, few models exist for training endoscopic retrograde cholangiopancreatography (ERCP) in patients with surgically altered anatomy. This training is necessary as the number of bariatric surgeries performed worldwide increases. ETM with human-like anatomy were developed to represent the postoperative anatomy after Billroth II (BII) reconstruction for a standard duodenoscope and the situs of a long-limbed Roux-en-Y (RY) for device-assisted enteroscopy (DAE). In three independent workshops, the models were evaluated by international ERCP experts. In RY model, a simulation for small bowel behavior in endoscopy was created. Thirty-three experts rated the ETM in ERCP expert courses. The BII model was evaluated as suitable for training (school grades 1.36), with a haptic and visual impression rating of 1.73. The RY model was rated 1.50 for training suitability and 2.06 for overall impression. Animal tissue-free ETMs for ERCP in surgically altered anatomy were successfully created. Evaluation by experienced endoscopists indicated that the models are suitable for hands-on ERCP training, including device-assisted endoscopy. It is expected that patient care will improve with appropriate training in advanced procedures.

## Introduction

The focus of endoscopy is changing from a pure diagnostic to a therapeutic and interventional tool. More patients with different postoperative anatomy are referred for endoscopic complication management due to increasing numbers of complex oncologic surgery and newly especially bariatric interventions^1^. A Roux-en-Y (RY) or a Billroth II (BII) reconstruction is a common option in upper gastrointestinal (GI) tract surgeries^2,3^. The necessity for endoscopic interventions is increasing, especially for patients after bariatric Roux-Y gastric bypass because of the high incidence of biliary stones after significant weight reduction^4^. Therefore, different approaches are available^5^. Knowing the altered anatomy and interpreting the anatomical situation for the endoscopist should be the first step. The second step, if necessary, is the training of device-assisted enteroscopy (DAE) for endoscopic retrograde cholangiopancreatography (ERCP). At the moment, single- and double balloon enteroscopy are possible options. Furthermore, laparoscopic guided trans-gastric and EUS-guided (endoscopic ultrasound) approaches are also available if a transpapillary access is too difficult^5,6^.

Training of new endoscopic techniques is important especially for technical developments and increasing procedural complexity. The European Society of Gastrointestinal Endoscopy (ESGE) has recognised this unmet need and stated that endoscopic techniques should be trained before being applied to patients^7,8^. Hence, training programs for DAE and ERCP have been introduced to improve endoscopists’ capabilities^9–12^. The 2019 Quality Improvement Publication “Performance measures for small bowel endoscopy: an ESGE Quality Improvement Initiative” stated that “According to an unpublished expert consensus on double-balloon enteroscopy (DBE), only advanced trainees should train in DAE,” and “[…] trainee proficiency should be assessed by direct observation of procedures before being signed off by their supervisor;”^7^. Ultimately, ESGE suggests in a guideline published in 2022 to use “[…] DAE-ERCP as a first-line endoscopic approach to treat pancreaticobiliary diseases in patients with surgically altered anatomy […]”^13^.

There is an unmet need for appropriate training opportunities for interventional endoscopy with altered anatomy. Investment in appropriate training methods positively affects complications and downtime the length of examinations^11^. This study aimed to develop and evaluate new training models for interventional endoscopy in patients with postoperatively altered anatomy. In particular, training in DAE in an animal material-free model should be enabled.

## Results

A 3D printed mold of the duodenum and a jejunal loop were constructed. The organs were created from the three molds and added to organ complexes along with a mold of the stomach. Intestinal texture was simulated by surface processing of the 3D-printed molds^14^.

### Evaluation of the BII model

The BII model was evaluated by fifteen participants (mean age 45 years, mean endoscopic experience 13 years). Each performed an average of 487 gastroscopies, 419 colonoscopies, 194 ERCPs, and 7 ERCPs on patients with altered postoperative anatomy per year (Table 1).

**Table 1:** Average (${\overline{\text{X}}}$) and standard deviation (σ) of the answers and their corresponding questions in the questionnaire for the BII (a) and the RY (b) model.

BII model				RY model			
1. How did you like the passage to the first anastomosis?		${\overline{\text{X}}}$	σ	1. How did you like the passage to the first anastomosis?		${\overline{\text{X}}}$	σ
1	How easy was it?	2.31	1.16	1	How easy was it?	1.95	1.05
2	How realistic was it?	2.00	0.89	2	How realistic was it?	2.16	1.14
2. How did you like the Brauns footpoint-anastomosis?				2. How did you like the second anastomosis?			
3	How easy was identifying the alimentary and the biliary limb?	2.44	0.86	3	How comparable was the view of the footpoint anastomosis to a real patient?	2.21	1.00
4	Was the situation comparable to a real patient?	1.94	0.75				
				3. How did you like the passage through the jejunum?			
				4	Did the haptics of the artificial mesenterium feel realistic?	2.16	0.99
				5	How comparable was using the balloon-enteroscope for the passage to a real patient?	2.17	0.90
3. How did you like the papilla?				4. How did you like the P papilla?			
5	How easy was the scope placement for the papilla?	2.63	0.93	6	How easy was the scope placement for the papilla?	2.32	0.57
6	Was the difficulty of scope placement comparable to a real patient?	2.13	0.93	7	Was the difficulty of scope placement comparable to a real patient?	2.37	0.57
7	How difficult was the cannulation of the papilla?	2.25	0.90	8	How difficult was the cannulation of the papilla?	2.16	0.63
8	Was the difficulty of the cannulation comparable to a real patient?	2.13	0.86	9	How important was the use of the balloon for the correct scope placement?	2.21	0.74
				10	Was the difficulty of cannulation comparable to a real patient?	2.68	1.17
4. What were your overall Impressions of the model?					5. What were your overall Impressions of the model?		
9	How was the Surface?	1.91	0.79	11	How was the Surface?	1.76	0.81
10	How was the Color?	1.55	0.66	12	How was the Color?	1.59	0.77
11	How was the Anatomy?	1.45	0.50	13	How was the Anatomy?	1.76	0.88
12	How were the Haptics (tactile impression)?	1.73	0.75	14	How were the Haptics (tactile impression)?	2.00	0.91
13	How do you evaluate the Tuebinger model for altered anatomy?	1.64	0.88	15	How do you evaluate the Tuebinger model for altered anatomy?	2.06	0.87
14	How suitable is the Billroth II model for teaching endoscopy in Billroth II anatomy?	1.36	0.48	16	How suitable is the long limb Roux-en-Y for teaching endoscopy in Roux-en-Y anatomy?	1.65	0.84
15	Do you think that training with suitable training models for ERCP should be done more regularly?	1.18	0.39	17	How suitable is the model for training balloon-assisted enteroscopy?	1.50	0.50
16	Do you think that training with suitable training models for ERCP should be included in the professional training of endoscopists?	1.18	0.39	18	Do you think that training with suitable training models for ERCP and DAE should be done more regularly?	1.24	0.73
17	How significant is the model to train ERCP in altered anatomy?	1.27	0.45	19	Do you think that training with suitable training models for ERCP and DAE should be included in the professional training of endoscopists?	1.35	0.84
				20	How significant is the model to train ERCP in altered anatomy?	1.53	0.78

The duration of the procedure varied between participants. The objective for the trainees during the workshop was successful cannulation of the papilla, which was not achieved by all participants.

The gastro-enterostomy was considered realistic (Question 2 in Table 1 (Q2), ${\overline{\text{X}}}$ = 2.00), and identifying the afferent and efferent limbs and aligning the endoscope in front of the papilla was rated easy to neutral (Q3 ${\overline{\text{X}}}$ = 2.44 and Q5 ${\overline{\text{X}}}$ = 2.63), similar to real patients (Q4 ${\overline{\text{X}}}$ = 1.94 and Q6 ${\overline{\text{X}}}$ = 2.13). The model was rated as realistic (Q9—Q12) and very suitable for training altered postoperative anatomy (Q14 ${\overline{\text{X}}}$ = 1.36) for the overall evaluation.

**Figure 1:**
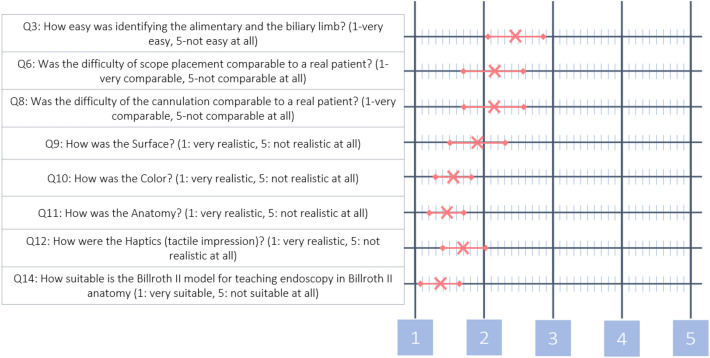
Ratings of mean value and standard deviation for key questions in mean in the questionnaire for the BII model.

Participants indicated that ERCP training should be more frequent for postoperatively altered anatomy using the BII model (Q15 ${\overline{\text{X}}}$ = 1.18) and that appropriate training tools, such as the present model, should be used during residency training (Q16 ${\overline{\text{X}}}$ = 1.29). Figure 1 shows a graphic display of the above-mentioned rating with average and standard deviation. Table 2 shows the point-by-point query with answers from the participants.

**Table 2:** Sociometric data and information on the training level of the participants who evaluated the BII-Model and the RY-Model.

BII model			RY model		
15 Participants	${\overline{\text{X}}}$ (Average)	σ (Standard deviation)	18 Participants	${\overline{\text{X}}}$ (Average)	σ (Standard deviation)
Age (years)	45	10	Age (years)	45	11
Time in Endoscopy (years)	13	6	Time in Endoscopy (years)	13	7
Number of performed endoscopies (n) per year					
Gastroscopies (n)	487	234	Gastroscopies (n)	475	258
Colonoscopies (n)	419	258	Colonoscopies (n)	443	255
ERCPs (n)	194	226	ERCPs (n)	183	46
ERCP with altered anatomy (n)	7	13	ERCP with altered anatomy (n)	10	19

### Evaluation of the RY model

The RY model was evaluated by eighteen participants (an average of 45 years and a mean of 13 years of endoscopic experience). The participants performed an average of 475 gastroscopies, 443 colonoscopies, 183 ERCPs, and 10 ERCPs on patients with altered postoperative anatomy per year (Table 1).

The time for the intervention varied for different participants between 5 and 15 min. Every participant was able to reach the papilla region. The use of the balloon overtube was mandatory in every intervention.

**Figure 2:**
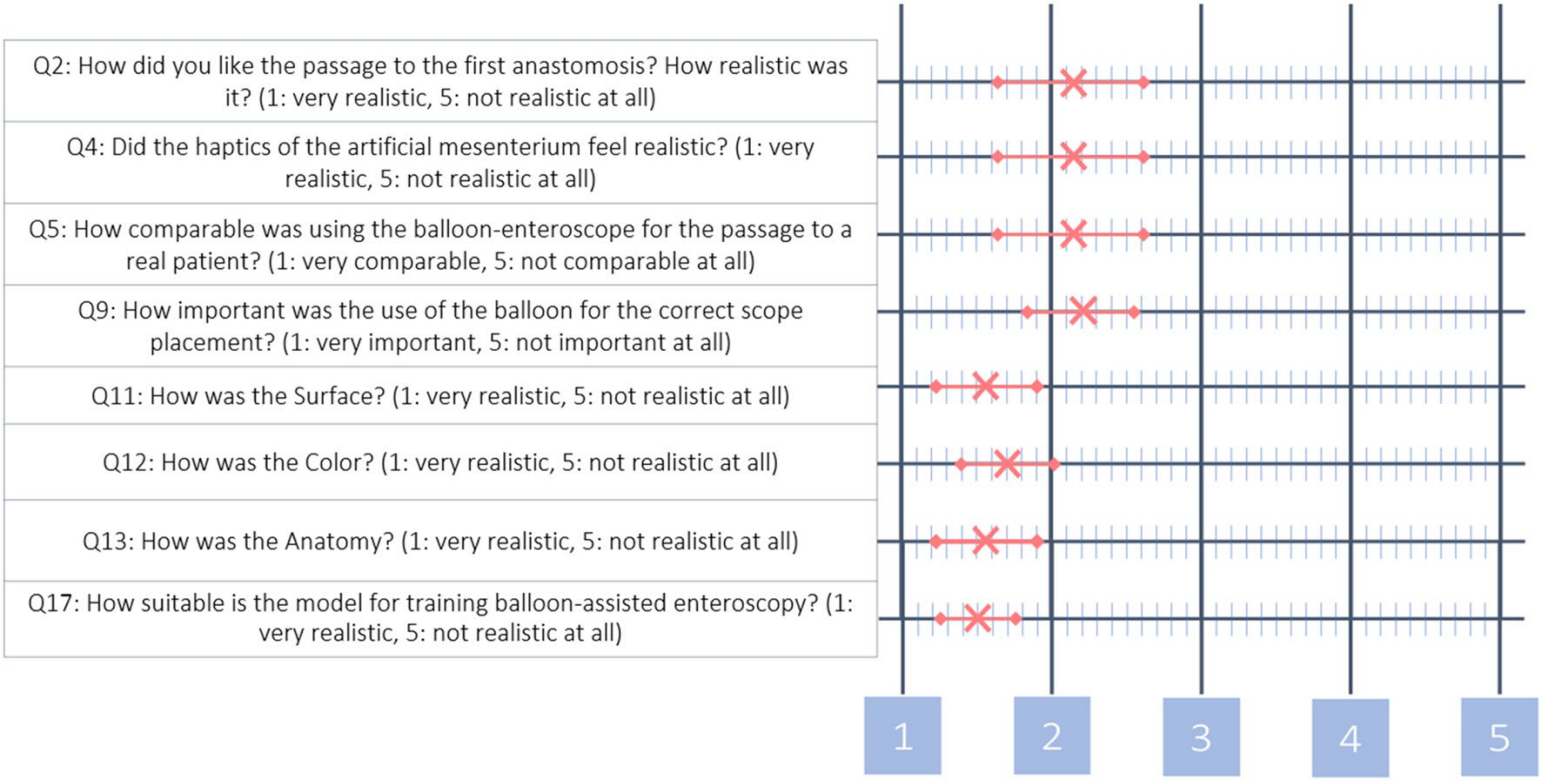
Ratings of mean value and standard deviation for key questions evaluated in the questionnaire for the Roux-en-Y model.

Participants rated both anastomoses as realistic and comparable (Q2 ${\overline{\text{X}}}$ = 2.16 and Q3 ${\overline{\text{X}}}$ = 2.21) as well as the tactile impression of the jejunal passage (Q4 ${\overline{\text{X}}}$ = 2.16), which was described as similar to real patients (Q5 ${\overline{\text{X}}}$ = 2.17). The model received good to very good ratings for surface (Q11 ${\overline{\text{X}}}$ = 1.76), color (Q12 ${\overline{\text{X}}}$ = 1.59), and anatomy (Q13 ${\overline{\text{X}}}$ = 1.76). The model was rated very suitable (Q16 ${\overline{\text{X}}}$ = 1.65) for training ERCP in long limb RY anatomy and for training DAE (Q17 ${\overline{\text{X}}}$ = 1.50). Participants strongly agreed (Q18 ${\overline{\text{X}}}$ = 1.24) with the necessity for suitable training models for ERCP and DAE that should be part of residency training (Q19 ${\overline{\text{X}}}$ = 1.35). Overall, the model was rated very significant for training ERCP in altered postoperative anatomy (Q20 ${\overline{\text{X}}}$ = 1.53). Figure 2 shows a graphic display of the above-mentioned rating with average and standard deviation. Table 2 includes the point-by-point query with participants’ answers.

## Discussion

The endoluminal approach for ERCP should be considered as a first step in patients with altered anatomy as it has the lowest risk of serious adverse events compared to EUS-guided or laparoscopic approaches^5,15^.

The safety and feasibility of medical interventions depend on the personal experience of the physicians; therefore, training with virtual reality (VR) and mechanical simulators has a significant impact on diagnostic and interventional endoscopy in particular for ERCP^11,16,17^. Biomodels are characterized by excellent surface properties for mucosal procedures. However, compared to human anatomy, the porcine cadavers commonly used in training differ significantly^18^. For procedures where hepatobiliary anatomy is critical, such as the exact dimensions of the duodenum and pathways to the papilla, this is a disadvantage for biomedical models. Certain VR models and computerized systems are promising for endoscopic training, but technical limitations make many of them unsuitable for complex procedures, but this could change^17^. There is limited data on VR models for expert training, and most VR systems remain costly. Artificial organ models increase the realism of training as they are compatible with standard endoscopes and devices. They also provide precise and reproducible anatomical features.

The ESGE has stated that specific DAE training is not available, although certification requires endoscopists to perform a certain number of ERCP procedures under supervision before starting DAE-ERCP^7,13^. To date, no training model allows realistic DAE training simulating a “real” patient. In a positional paper of the OECD on “The economics of patient safety […]”, calculations suggest that investing in demand-driven training structures will, with high probability be lower than the costs of possible complication management^19^. Bio-models have good surface properties that are useful and suitable for mucosal interventions^10^. Unfortunately, the haptic properties and the duodeno-pancreatic anatomy significantly differ from “real” patients^18^. Navigating non-standard anatomy is an ongoing challenge for endoscopists despite the growing demand^1,20^. Therefore, training ERCP for patients with surgically altered anatomy requires realistic artificial models. To date, no training model allows realistic DAE training simulating a “real” patient. Participants of the three workshops for advanced ERCP were unanimous in their opinion that suitable DAE-ERCP training models are required and should be included in clinical training. These results revealed a high, unmet demand for this type of model because DAE-ERCP could not be trained in patient analog models.

The novel duodenoscope (BII model) and DAE (RY model) ETM were evaluated by experienced endoscopists. Specifically, we focused on orientation and the ease of pathway identification. The Whipple procedure or omega-loop bypass in bariatric surgery are also common procedures although BII reconstructions are rarely performed. Overall, the BII model demonstrated that interventions were trainable and that papillary intubation could be realistically simulated.

The RY model is the first ETM as we know for DAE with a target structure/papilla. Therefore, tactile impression evaluation of the artificial mesenterium and balloon enteroscopy is essential for the model’s suitability. As the model is built openly, the endoscopist can have a view over the moving small bowel and see the behavior of the endoscope like in fluoroscopy, without the need for radiation. The experienced participants rated the balloon’s behavior in the artificial jejunum as realistic.

The question of whether training on the ETMs presented actually leads to a higher success rate of transpapillary ERCP in patients with altered anatomy cannot be answered by the data presented. A comparable model, such as Frimberger et al.'s training model from 2008 and 2016, initially assessed comparability to real anatomical situations and approaches solely through expert opinions^21,22^. Subsequently, a follow-up study demonstrated a training effect among participants, showcasing improvements in examination times and handling skills, reinforcing the evidence base^11^. A study with a parallel design could be pursued for the current model. However, it's important to note that the evidence in this study relies on expert opinions. Follow-up studies are needed to analyse the long-term effect of ETM training and reinforce this evidence.

In literature the technical success rates of reaching the papilla in altered anatomy patients with Single Balloon Enteroscopy (SBE) ranges from 70 to 85%^23–26^. Although there are comparative studies between DAE, they lack the primary endpoint of papillary access for ERCP^27–29^. Since no comparable data is available, ERCP in patients with altered anatomy is performed using the endoscopist’s preferred technique^5^. The key factors in DAE confidence include feasible training, routine, and experience, including training models. Not all endoscopists experienced in ERCP are necessarily well trained in DAE and vice versa. For DAE-ERCP endoscopist should be trained in both^7,13^. As seen in the demographic data (Table 1), altered anatomy ERCP cases are still rare in comparison to normal ERCPs, which leads to a lack of experience and patient safety.

The technical limitations of the model mainly concern the increased resistance between the rubber surface of the endoscopes and the inner wall of the latex organs. Therefore, sufficient lubricant must be used at the beginning of the examination. Silicon spray and lubricant were used during interventions, which decreased friction with every intervention. The lack of peristalsis, which is a significant challenge in real patients, is another limitation of mechanical artificial models. Peristalsis is more easily achieved using VR systems. Further, the introduced mechanical models are not ready for a pressure-controlled system where the risk of endoscopic perforation by mechanical forces could be monitored. Typically, the latex structures are very elastic and therefore do not resemble the small bowel’s mechanical properties. Specifically, we cannot reliably detect accidental perforations by strong forces during enteroscopy so far.

To the best of our knowledge, this is the first description of training models in postoperative altered anatomy and DAE. Training with such models, as demonstrated in the manuscript, could improve the handling of difficult situations/anatomies, even for experts.

To the best of our knowledge, this is the first description of training models in postoperative altered anatomy and DAE. Friction and peristaltic challenges should be considered in future endoscopic training protocols. However, the range of endoscopic training capabilities required by ESGE, apart from real patients, could be met for the applications described. Training with such models, as demonstrated in the manuscript, could improve the handling of difficult situations/anatomies, even for experts. Thus, the models could be used and established as a building block of a larger structural training approach.

## Methods

### BII model

**Figure 3:**
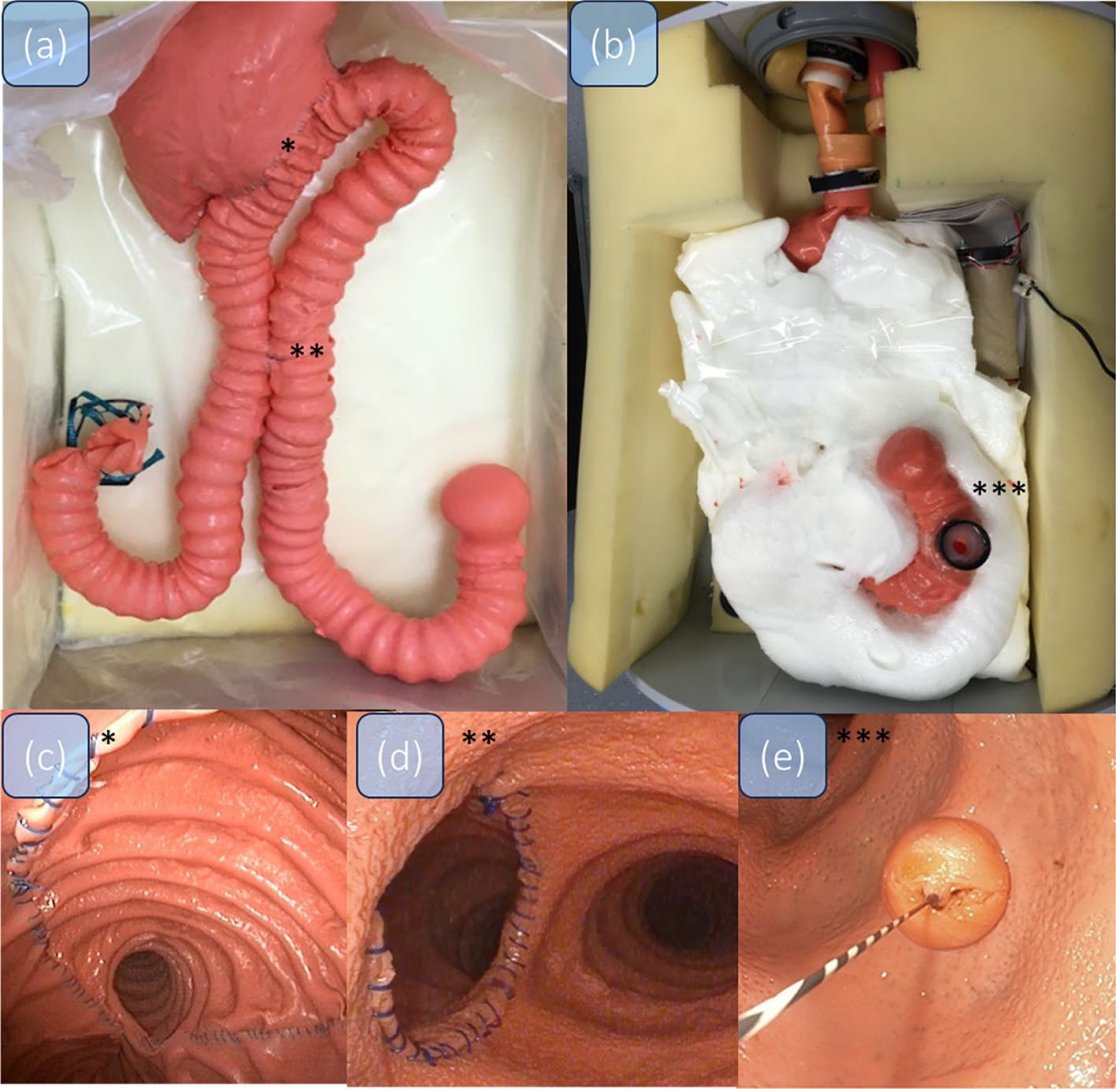
BII (**a**) Overview of the anatomy of the BII model; * marks the gastroenterostomy, ** marks the jejuno-jejunostomy, *** marks the papilla. (**b**) The finished, foamed Inlay of the BII model with the built-in papillary system. (**c**) Endoscopic view inside of the gastroenterostomy, side-to-side gastrojejunostomy (**d**) and the papilla with a guide wire in place (**e**).

The BII reconstruction after gastric resection contains a side-to-end gastro-jejunostomy and a Braun’s footpoint anastomosis (jejuno-jejunostomy). Figure 3a shows an overview over the anatomy in the BII anatomy, Fig. 3b shows the finished inlay of the model. The entire endoscopic training model (ETM) (Fig. 3a), contents on a head, oesophagus, gastric pouch with gastrojejunostomy (Fig. 3c) and jejuno-jejunostomy (Fig. 3d) with possible retrograde passage to the duodenum with papilla (Fig. 3e). The total length of the BII model is 140 cm from the artificial mouth to the papilla. The model allows sphincterotomy, lithotripsy, and multiple different bile duct interventions (Fig. 3e)^30^. For an endoscopic live video of the model, see Video 1^14^.

### RY model

**Figure 4:**
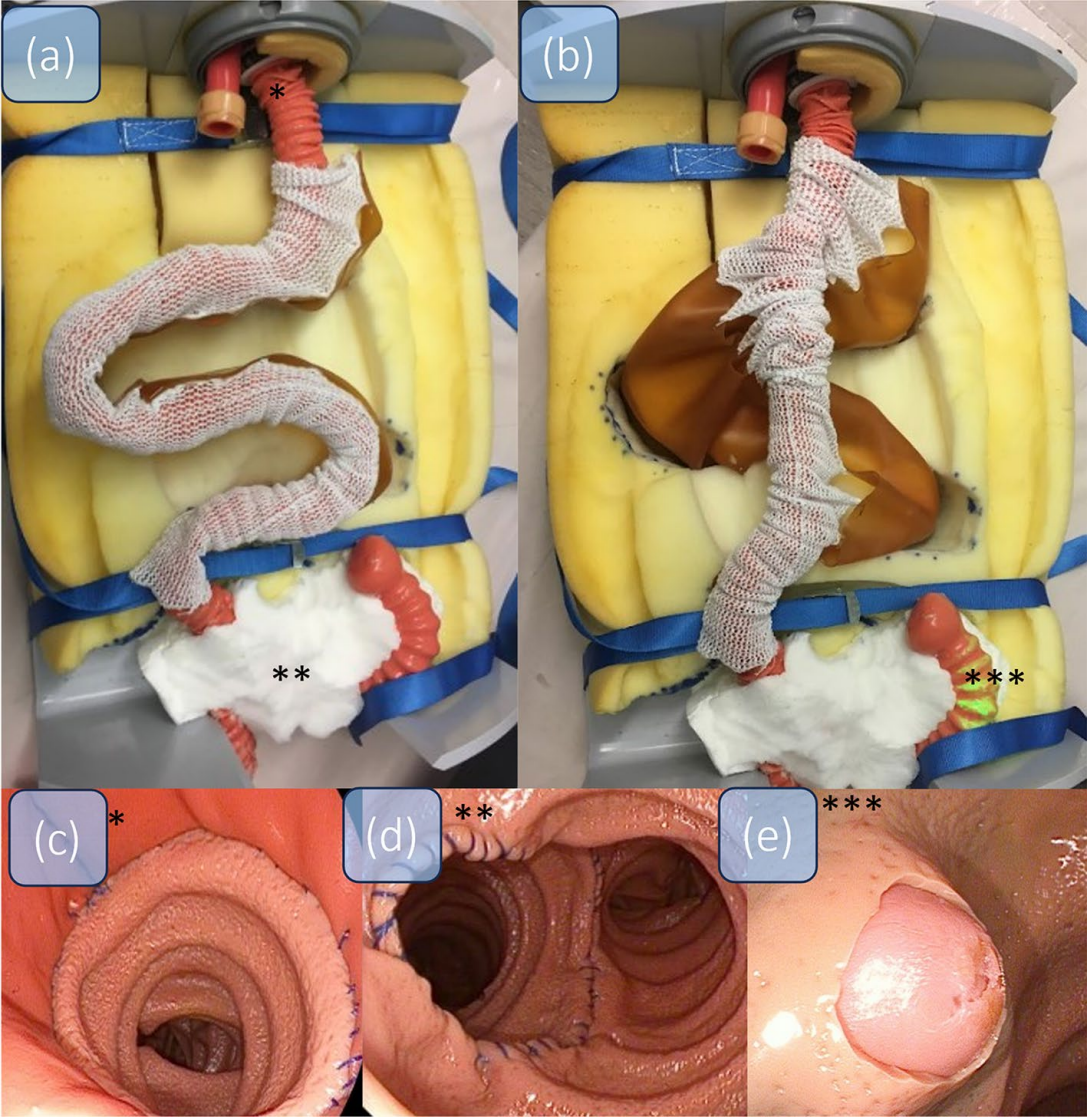
RY (**a**) Overview of the RY-model; * marks the gastrojejunostomy, ** marks the jejuno-jejunostomy, *** marks the papilla. In (**b**), with the use of the balloon enteroscope, the loops are straightened and shortened. In (**c**) and (**d**) the endoscopic view of the gastrojejunostomy (**c**) and the jejunojejunostomy (**d**) are displayed. In (**e**) the endoscopic view of the papilla is shown.

The long limb RY is an intended consequence of the bariatric RY gastric bypass procedure. The RY-ETM contents on a head, esophagus, gastric pouch (Fig. 4c), the gastrojejunostomy, and the jejunojejunostomy after 100 cm (Fig. 4d). The 100 cm of small bowel contains many loops, hangs loosely in the artificial mesentery and requires the use of a balloon enteroscope. Insufflation of the balloon and subsequent push and pull manoeuvres straighten and shorten the tortuous part of the small bowel (Fig. 4, see comparison between (a) and (b)). The total length of the RY model is 190 cm from the artificial mouth to the papilla. For a live endoscopic video of the model, see Video 2^14^.

### Model development

The models are based on existing endoscopy training models known as “Tübinger models”^31^. Two models with altered postoperative anatomy were created using “Blender V.279” software and analog data from patients’ data sets. Three-dimensional (3D)-printed polylactic acid molds for the stomach, duodenum, and jejunum were immersed in a latex solution, dried, and then anastomosed using a continuous suturing technique. The organs were firmly reconstructed and fixed in the retroperitoneum, and the situs was embedded in polyurethane (PU) foam. The small intestine from Treitz is suspended using an “artificial mesentery” without PU foam fixation^14^. The models have a modular design. The individual organ-systems are assembled using plug-in connections. This modular design allows to easily attach different stomachs with modified anatomy to the same esophagus. Thus, a whole range of altered anatomy can be represented in a full model with little effort.

### Evaluation

The Ethics Committee of the University Hospital of Tübingen (735/2019BO2; date of approval: December 04, 2019) approved the model evaluation, which was conducted at three independent international advanced ERCP training workshops in February 2020 and June 2023. The evaluation was carried out in accordance with the Declarations of Helsinki. Informed consent was obtained from all participants. The evaluation is based on anonymous questionnaires according to 17 questions for the BII model, and 20 questions for the RY model. The questionnaire was designed on the basis of representative questionnaires of comparable models or models with similar characteristics^23,32^. The response options were (1) very good/easy/comparable/realistic; (2) good/easy/comparable/realistic; (3) neutral; (4) not good/easy/comparable/realistic; (5) not good/easy/comparable/realistic at all. The advanced participants were asked to compare the model with conditions in real patients from their own experience.

Table 1 shows the questions. A duodenoscope (TJF-Q190V, Olympus) was used for the BII model, and a single-balloon enteroscope (SIF-H190, Olympus) with a single-use balloon Overtube (ST-SB1, Olympus) was used for the RY model evaluations. Silicon spray was used to reduce friction. Fifteen participants evaluated the BII model and eighteen the RY model. Table 2 shows the socio-demographic and educational data of the participants.

### Statistics

The descriptive statistic was conducted with Excel 2019 for Windows 10 (MS Office).

### Supplementary Information

Supplementary Legends.

Supplementary Video 1.

Supplementary Video 2.

## Data Availability

The datasets generated during and/or analysed during the current study are available from the corresponding author on reasonable request.

## References

[1] English WJ (2018). American Society for Metabolic and Bariatric Surgery estimation of metabolic and bariatric procedures performed in the United States in 2016. Surg. Obes. Relat. Dis..

[2] Roux, C. De la gastro-enterostomie, Revue de gynecologie et de chirurgie abdomninale I. 67 (1897).

[3] Olbe, L. & Becker, H. D. In *Magenchirurgie: Indikationen, Methoden, Komplikationen* (eds Becker, H. D., Lierse, W. & Schreiber, H. W.) 50–70 (Springer, 1986).

[4] Guzmán HM (2019). Incidence and risk factors for cholelithiasis after bariatric surgery. Obes. Surg..

[5] Moreels TG (2017). Techniques for endoscopic retrograde cholangiopancreatography in altered gastrointestinal anatomy. Curr. Opin. Gastroenterol..

[6] Dietrich CF (2022). How to perform EUS-guided biliary drainage. Endosc. Ultrasound.

[7] Spada C (2019). Performance measures for small-bowel endoscopy: A European Society of Gastrointestinal Endoscopy (ESGE) Quality Improvement Initiative. Endoscopy.

[8] Testoni PA (2016). Papillary cannulation and sphincterotomy techniques at ERCP: European Society of Gastrointestinal Endoscopy (ESGE) Clinical Guideline. Endoscopy.

[9] Yamamoto H (2015). Double-balloon endoscopy is safe and effective for the diagnosis and treatment of small-bowel disorders: Prospective multicenter study carried out by expert and non-expert endoscopists in Japan. Dig. Endosc..

[10] Hochberger J (2005). Training with the compactEASIE biologic endoscopy simulator significantly improves hemostatic technical skill of gastroenterology fellows: A randomized controlled comparison with clinical endoscopy training alone. Gastrointest. Endosc..

[11] Liao WC (2013). Coached practice using ERCP mechanical simulator improves trainees' ERCP performance: A randomized controlled trial. Endoscopy.

[12] Zhang C, Yuan Y, Qiu C, Zhang W (2014). A meta-analysis of 2-year effect after surgery: Laparoscopic Roux-en-Y gastric bypass versus laparoscopic sleeve gastrectomy for morbid obesity and diabetes mellitus. Obes. Surg..

[13] Pennazio M (2023). Small-bowel capsule endoscopy and device-assisted enteroscopy for diagnosis and treatment of small-bowel disorders: European Society of Gastrointestinal Endoscopy (ESGE) Guideline-Update 2022. Endoscopy.

[14] Koch, K. *Entwicklung von Trainingsmodellen in der flexiblen Endoskopie - Herausforderung der veränderten postoperativen Anatomie* Dissertation thesis (University of Tuebingen, 2023).

[15] Kamal F (2023). Efficacy and safety of EUS-guided biliary drainage for benign biliary obstruction—A systematic review and meta-analysis. Endosc. Ultrasound.

[16] Cheng CL (2006). Risk factors for post-ERCP pancreatitis: A prospective multicenter study. Am. J. Gastroenterol..

[17] Zhang W, Liu X, Zheng B (2021). Virtual reality simulation in training endoscopic skills: A systematic review. Laparosc. Endosc. Robot. Surg..

[18] von Trotha KT (2015). Vascular anatomy of the small intestine—A comparative anatomic study on humans and pigs. Int. J. Colorectal Dis..

[19] Slawomirski L, Auraaen A, Klazinga NS (2017). The economics of patient safety: Strengthening a value-based approach to reducing patient harm at national level. OECD Health Working Pap..

[20] Welbourn R (2019). Bariatric surgery worldwide: Baseline demographic description and one-year outcomes from the fourth IFSO global registry report 2018. Obesity Surg..

[21] Frimberger E, Abdelhafez M, Schmid RM, von Delius S (2016). A novel mechanical simulator for cannulation and sphincterotomy after Billroth II or Roux-en-Y reconstruction. Endosc. Int. Open.

[22] Frimberger E (2008). A novel and practicable ERCP training system with simulated fluoroscopy. Endoscopy.

[23] Shimatani M, Takaoka M, Ikeura T, Mitsuyama T, Okazaki K (2014). Evaluation of endoscopic retrograde cholangiopancreatography using a newly developed short-type single-balloon endoscope in patients with altered gastrointestinal anatomy. Dig. Endosc..

[24] Kawamura T (2015). Clinical usefulness of a short-type, prototype single-balloon enteroscope for endoscopic retrograde cholangiopancreatography in patients with altered gastrointestinal anatomy: Preliminary experiences. Dig. Endosc..

[25] Tanisaka Y (2023). Single-balloon enteroscopy-assisted endoscopic retrograde cholangiopancreatography in patients with surgically altered anatomy: A technical review. Korean J. Gastrointest. Endosc..

[26] Yane K (2017). Short-type single-balloon enteroscope-assisted ERCP in postsurgical altered anatomy: Potential factors affecting procedural failure. Endoscopy.

[27] Frieling T, Heise J, Sassenrath W, Hülsdonk A, Kreysel C (2010). Prospective comparison between double-balloon enteroscopy and spiral enteroscopy. Endoscopy.

[28] Lenz P, Domagk D (2012). Double- versus single-balloon versus spiral enteroscopy. Best Pract. Res. Clin. Gastroenterol..

[29] May A, Manner H, Aschmoneit I, Ell C (2011). Prospective, cross-over, single-center trial comparing oral double-balloon enteroscopy and oral spiral enteroscopy in patients with suspected small-bowel vascular malformations. Endoscopy.

[30] Schweizer, U. *Optimiertes Hands-on-Trainingsphantom für die ERCP | Evaluation und Vermeidung von Strahlenbelastung* Dissertation thesis (University of Tuebingen, 2023).

[31] Grund KE, Schweizer U, Zipfel A, Mothes B (2018). Learning of flexible endoscopy, particularly endoscopic vacuum therapy (EVT). Chirurg.

[32] Cooper L, Sindali K, Srinivasan K, Jones M, Nugent N (2019). Developing a three-layered synthetic microsurgical simulation vessel. J. Reconstr. Microsurg..

