# Supplementary material for: Development and evaluation of artificial organ models for ERCP training in patients with surgically altered anatomies
Source: Sci Rep. 2023 Dec 21;13:22920. doi: 10.1038/s41598-023-49888-3 (PMC10739860; doi:10.1038/s41598-023-49888-3)
Supplement: Supplementary file 1 — Supplementary Legends. [file 41598_2023_49888_MOESM1_ESM.docx]

**Video 1:** Video 1 shows the BII model. The endoscope starts at the Braun's anastomosis and is retracted through the alimentary limb into the stomach. The gastroenterostomy is shown as the endoscope enters the stomach. After passing through the gastroenterostomy into the biliary limb, the view of the Braun's anastomosis can be seen again.

**Video 2:** Video 2 shows the RY model in retraction. At the beginning you can see the papilla as the endoscope is retracted. The endoscope passes the anastomosis and enters the pouch after rapid retraction.
